# Coverage of Megaprosthesis with Human Acellular Dermal Matrix after Ewing's Sarcoma Resection: A Case Report

**DOI:** 10.1155/2011/978617

**Published:** 2011-07-25

**Authors:** Robert M. Whitfield, Jeremy Rinard, David King

**Affiliations:** ^1^Department of Plastic Surgery, Medical College of Wisconsin, 8700 Watertown Plank Road, Milwaukee, WI 53226, USA; ^2^Department of Orthopaedic Surgery, Medical College of Wisconsin, 8700 Watertown Plank Road, Milwaukee, WI 53226, USA

## Abstract

A 23-year-old female with Ewing's Sarcoma underwent tibial resection and skeletal reconstruction using proximal tibial allograft prosthetic reconstruction with distal femur endoprosthetic reconstruction and rotating hinge. Human acellular dermal matrix, (Alloderm, LifeCell, Branchburg, NJ, USA), was used to wrap the skeletal reconstruction. Soft tissue reconstruction was completed with a rotational gastrocnemius muscle flap and skin graft. Despite prolonged immobilization, the patient quickly regained full range of motion of her skeletal reconstruction. Synthetic mesh, tapes and tubes are used to perform capsule reconstruction of megaprosthesis. This paper describes the role of human acellular dermal matrix in capsule reconstruction around a megaprosthesis.

## 1. Introduction

Megaprosthesis reconstruction in combination with large soft tissue resection leaves complicated wounds at increased risk for infection. Patients will be immobilized in full extension following reconstruction of the extensor mechanism to minimize the risk of wound complications and optimize active knee extension following reconstruction of the knee with megaprosthesis. The trevira tube, Dacron, and other synthetic meshes have been used in the reconstruction of joint capsule and reattachment of muscles [1, 2]. These synthetic products do not completely isolate the prosthesis within the wound. Another way to perform the capsule reconstruction is to use human acellular dermal matrix, Alloderm (LifeCell, Branchburg, NJ, USA). Near-complete isolation of the megaprosthesis can be achieved with this reconstructive technique. The acellular dermis has had the cells removed through chemical and physical processing [3]. 

This leaves a biologic scaffold capable of cellular in-growth and revascularization [4]. 

This paper demonstrates the use of human acellular dermal matrix in the capsule reconstruction around a megaprosthesis.

## 2. Materials and Methods

**Figure 1:**
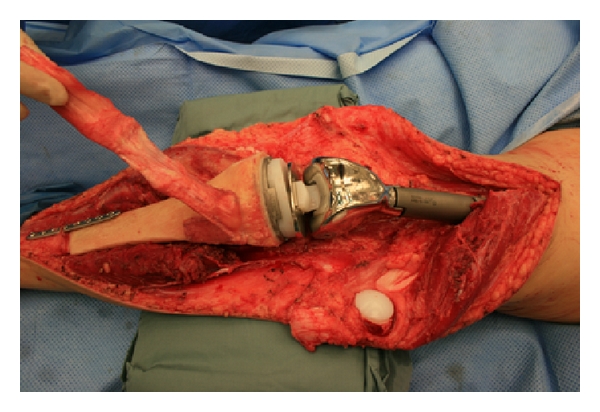
Skeletal reconstruction.

**Figure 2:**
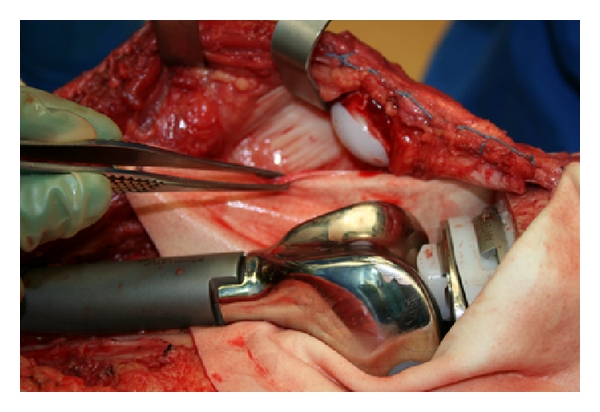
Attachment of alloderm to residual capsule and border of patellar tendon.

**Figure 3:**
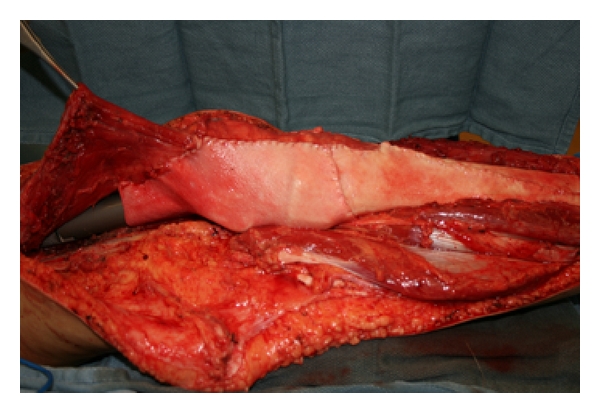
Wrapping of megaprosthesis.

**Figure 4:**
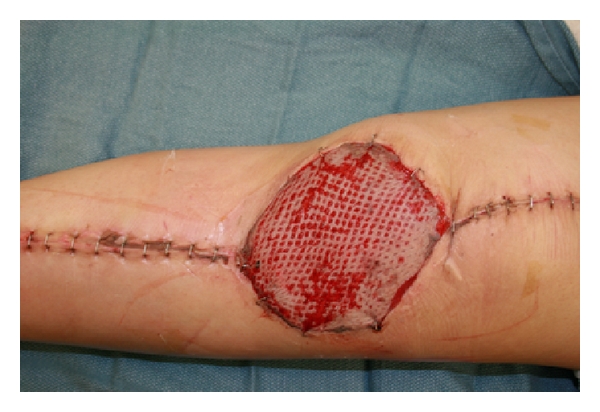
Gastrocnemius flap and split thickness skin graft at 1 week.

A 23-year-old female with Ewing's sarcoma of left lower extremity underwent tumor resection, immediate skeletal reconstruction, knee capsule reconstruction, and soft tissue reconstruction. The resection was a wide resection of distal femur and proximal tibia with preservation of the extensor mechanism. Skeletal reconstruction was completed using proximal tibial allograft prosthetic reconstruction with distal femur endoprosthetic reconstruction and rotating hinge (Figure 1). The extensor mechanism had been reconstructed using the allograft extensor tendon oversewn to the native extensor tendon remnant. The acellular dermis was wrapped and around the prosthesis to set the tension. After setting the tension, the acellular dermis was sutured to the medial border of the patellar tendon (Figure 2). An additional sheet of acellular dermis was sutured to the previously placed acellular dermis to completely wrap the tibial allograft (Figure 3). The patient was placed in 15° of flexion, and the coverage of the remaining prosthesis was completed by suturing the acellular dermis to the lateral border of the quadriceps tendon. The reconstruction was then evaluated by ranging the skeletal reconstruction to evaluate the integrity of the capsular reconstruction. After the megaprosthesis had been wrapped by acellular dermal matrix, the soft tissue reconstruction was performed using a rotational gastrocnemius muscle flap with a split thickness skin graft (Figure 4).

## 3. Results

By using acellular dermal matrix, a more complete capsular reconstruction was performed in this patient. After being immobilized for 6 weeks, the active ROM for flexion was patient 70° of active flexion. The patient was placed into physical therapy with weight bearing as tolerated in a knee immobilizer. At three months postoperation the patient had active ROM to 120° on the left with no extensor lag. The patient was able to ambulate without any assistive devices. Her wound had healed without any complications.

## 4. Discussion

Acellular dermal matrix is a regenerative matrix that allows for tissue ingrowth. When placed in contaminated wounds of the abdomen, it allows for healing, helping to salvage severely ill patients lives [3, 5]. Human acellular dermal matrix rapidly became incorporated into abdominal wall reconstruction but has fallen out of favor due to the fact that even when set under significant tension, it stretches [6]. However, acellular dermis that has integrated shows great capacity to avoid dehiscence at the interface with native tissues [7]. Capsular contracture is a significant problem in implant-based breast reconstruction, but with the introduction of acellular dermal matrix into prosthetic breast surgery, capsular contracture has decreased significantly [8, 9]. In prosthetic breast surgery, this appears to be due to the ability of acellular dermis to decrease the inflammatory response in capsule formation [10]. These properties make acellular dermal matrix an attractive material in capsular reconstruction. In the case presented, the patient had been immobilized for several weeks. Despite this long period of immobilization, the active ROM for flexion was 120°. In a study with similar patients, the average active ROM for flexion was 85.5°. Due to the ability of acellular dermal matrix to stretch, avoid dehiscence, and limit capsular contracture, it is another material to consider in megaprosthesis reconstruction.
